# Psychological and Behavioral Impact of Participation in Ovarian Cancer Screening

**DOI:** 10.3390/diagnostics7010015

**Published:** 2017-03-08

**Authors:** Michael A. Andrykowski

**Affiliations:** Department of Behavioral Science, University of Kentucky College of Medicine, Lexington, KY 40536-0086, USA; mandry@uky.edu

**Keywords:** ovarian cancer, psychological, psychosocial, behavioral, screening, false positive test result

## Abstract

Evaluation of costs and benefits associated with cancer screening should include consideration of any psychological and behavioral impact associated with screening participation. Research examining the psychological and behavioral impact of screening asymptomatic women for ovarian cancer (OC) was considered. Research has focused upon potential negative psychological (e.g., distress) and behavioral (e.g., reduced future screening participation) impact of false positive (FP) OC test results. Results suggest FP OC screening results are associated with greater short-term OC-specific distress. While distress dissipates over time it may remain elevated relative to pre-screening levels for several weeks or months even after clinical follow-up has ruled out malignancy. The likelihood of participation in future OC screening may also be reduced. Research focused upon identification of any beneficial impact of participation in OC screening associated with receipt of “normal” results was also considered. This research suggests that a “normal” screening test result can have psychological benefits, including increased positive affect and beliefs in the efficacy of screening. It is concluded that any psychological or behavioral harms attributable to OC screening are generally very modest in severity and duration and might be counterbalanced by psychological benefits accruing to women who participate in routine OC screening and receive normal test results.

## 1. Introduction

For some cancers, participation in cancer screening can lead to early diagnosis and an associated improvement in five-year survival. Consequently, great effort has been expended to develop cost-effective screening tests and protocols for cancers of the breast, colon, rectum, cervix, prostate, lung, and ovary—cancers with a high likelihood of treatment success when diagnosed and treated at an early stage of disease. Furthermore, for those screening tests for which the evidence suggests cost-effectiveness, great effort has been expended to ensure widespread uptake and appropriate repeat screening by screening-eligible individuals.

While participation in cancer screening can improve prognosis for some cancers, cancer screening is not without its drawbacks. All cancer screening approaches yield some proportion of inconclusive or abnormal results. These results typically require additional clinical follow-up to determine if a malignancy is present. Clinical follow-up might include surgery or biopsy, performance of a second-line screening test, or perhaps simply a repeat of the original screening test. In most instances, follow-up indicates the original abnormal or inconclusive screening test result is benign—no malignancy is present. Such false positive (FP) results may not be psychologically or behaviorally benign, however. A survey of recipients of FP test results in the context of breast, prostate, cervical or colorectal cancer screening found 40% described the experience as “very scary” or “the scariest time of my life” [1]. In general, research has shown a FP cancer screening test result can negatively impact both psychological (e.g., distress) and behavioral (e.g., participation in future cancer screening) outcomes [2,3,4,5,6], although research suggesting no impact is also available (e.g., [7]). 

Recommendation of any cancer screening test for routine use in asymptomatic, average-risk women is based on the relative balance of the benefits and costs associated with that screening test. Identification of the benefits of any cancer screening test is relatively straightforward. Whether a cancer screening test results in a significant reduction in the number of deaths due to that specific cancer (i.e., cancer-specific mortality) is the primary determinant of the benefits of a particular screening test. A broader view of the benefits of a screening test could include consideration of any psychological or behavioral benefits associated with a screening test. Though rarely considered, participation in cancer screening may yield positive outcomes including reductions in cancer worry or increases in feelings of reassurance and well-being [8,9].

As one might expect, calculation of the costs of a screening test include consideration of the monetary costs associated with testing. In addition, calculation of the costs of a screening test should also include the “costs” associated with FP screening test results. These costs include the monetary as well as physical morbidity costs associated with performance of additional follow-up procedures or unnecessary surgeries. In addition, a FP screening test result might also exact certain psychological or behavioral costs. Psychological costs include fear or anxiety, a heightened sense of personal risk for cancer, or reduced confidence in the efficacy of the screening test, all of which might result in a significant behavioral cost—a lessened likelihood of returning for repeat screening in the future. If the collective costs associated with a screening test exceed the collective benefits of screening, one might well conclude that a particular screening test does more harm than good.

The purpose of this paper is to consider the literature regarding the psychological and behavioral impact of participation in routine screening for ovarian cancer (OC). Evidence regarding the potential for OC screening to yield both positive and negative psychological and behavioral outcomes will be considered. 

## 2. Screening for Ovarian Cancer

When diagnosed and treated at a localized stage, OC is associated with a good prognosis. The five-year relative survival rate is 92% [10]. Unfortunately, the majority of cases of OC (61%) are diagnosed with late stage disease where existing treatment approaches are less likely to be successful and five-year relative survival rates are correspondingly only 27% [10]. Given this state of affairs, considerable effort has been expended to develop cost-effective approaches to screening for OC [11,12,13,14,15,16]. For the most part, these approaches include transvaginal ultrasonography (TVS) and serum tests for cancer antigen 125 (CA125), alone or in combination. While demonstrating some value in promoting early detection of OC in average-risk, asymptomatic women, no approach to OC screening has been shown to significantly reduce OC-specific mortality in asymptomatic, average-risk women in a prospective, randomized trial. As a result, implementation of routine screening for OC in asymptomatic, average-risk women has been controversial. Currently, the US Preventive Services Task Force recommends against routine screening for OC in asymptomatic, average-risk women (D recommendation) [17]. However, despite this recommendation, screening for OC is widely available and utilized for asymptomatic women at either average or elevated risk for OC. A recent survey of primary care physicians and obstetrician-gynecologists in the USA found many (33%) believed TVS and CA125 were effective screening tests for OC and many would offer OC screening tests to low (28%) and medium risk (65%) women [18]. 

## 3. Impact of False Positive OC Screening Test Results

Similar to screening tests for other cancers, screening for OC yields a certain proportion of inconclusive or abnormal test results [19]. For asymptomatic women at average risk for OC, approximately 5%–10% of OC screening tests yield an inconclusive or abnormal result, necessitating clinical follow-up. Fortunately, the vast majority of inconclusive or abnormal OC screening test results are ultimately deemed benign. While clinically benign, however, a FP OC screening test result may not be psychologically or behaviorally benign. An inconclusive or abnormal OC test result requiring additional clinical follow-up could understandably cause a woman to consider the possibility of a diagnosis of OC, resulting in a heightened sense of personal vulnerability. This in turn could result in the experience of fear, anxiety, and/or distress as well as a reduced likelihood of returning for future OC screening. 

What is the evidence for such reactions? To answer this question a group of 10 studies are considered [20,21,22,23,24,25,26,27,28,29]. While an attempt was made to use PubMed to identify all relevant studies, a systematic review of the literature was not conducted and consequently no guarantee is made regarding the completeness of the studies considered here. Each of the 10 studies considered involves comparison of two groups of women participating in OC screening: women receiving a “normal” screening test result (i.e., Normal group) and women receiving an abnormal test result with clinical follow-up revealing no malignancy present (i.e., FP group). Comparison of these two groups of women has the potential to shed light on the impact of a FP OC screening test result. The specific OC screening test(s) employed varies across these studies but each employed CA125 testing or ultrasonography, typically transvaginal sonography (TVS), either alone or in combination. The psychological and behavioral outcomes the Normal and FP groups are compared upon vary widely across studies. The most common outcomes being generic mental health outcomes (e.g., state anxiety, depression, distress), OC-specific measures of distress and worry, OC risk perception, and participation in future cancer screening, either actual or stated intention to participate. These studies also vary widely with regard to study design (e.g., cross sectional vs. longitudinal), sample size in both the Normal and FP groups, and the timing of the assessment of outcomes (e.g., short-term vs long-term). Finally, some of these studies focused upon OC screening for women at intermediate or high risk for OC, typically due to a strong family history of OC [23,25,26,27,28]. The remaining studies focused upon OC screening among asymptomatic women generally at average risk for OC [20,21,22,24,29].

### 3.1. Impact of FP Results on Women at Intermediate or High Risk for OC

In an early study, 266 women at risk for familial OC underwent either transabdominal or transvaginal ultrasonagraphy with color Doppler imaging [27]. Women with abnormal results on initial scan returned in six weeks for a repeat scan. Women with repeat abnormal results at the repeat scan were referred for surgery. All women completed a questionnaire 6–15 weeks before the initial scan and again after their initial scan. Women also completed the same questionnaire after a repeat scan. Psychological outcomes assessed included OC risk perception, cancer worry, coping style, depression and anxiety symptoms, and general distress. Results indicated that following an initial scan, before a repeat scan ruled out the presence of malignancy, women receiving an abnormal result (*n* = 51) reported greater cancer worry, general distress, and anxiety than women receiving a normal scan result (i.e., Normal group; *n* = 189). Of women receiving an abnormal result at the initial scan, 32 received a normal result at the repeat scan and thus constituted a FP group. In the FP group, distress returned to baseline levels but remained elevated relative to the Normal group. Overall, it was concluded FP results are associated with increased distress in the short-term but this adverse impact is neither severe nor persistent. 

Participants in this initial study completed the same study questionnaire as in the initial study one year after their initial scan [28]. In general, one year after their initial scan, women in the FP group did not differ from women in the Normal group with regard to distress and anxiety. However, more women in the FP group described themselves as “more worried” about cancer than women in the Normal group. It was concluded while FP results may be associated with some increased worry about cancer in the longer term, there is little evidence to suggest severe and persistent adverse psychological effects. 

Kauff et al. examined women at intermediate risk for OC due to a personal or family history of breast and/or ovarian cancer but no documented BRCA1/2 mutation [25]. Women were offered semi-annual OC screening using TVS, CA125 testing, and pelvic examination. Women completed a baseline assessment at enrollment in the screening program and follow-up assessments every six months thereafter in conjunction with OC screening. Impact on mental quality of life (QOL) was indexed by the Mental Component Score of the Medical Outcome Study Short Form-36. At least one follow-up assessment was completed by 135 women. During a mean of 19.8 months of follow-up, 52 women experienced ≥1 abnormal OC screening test result. None of these women were ultimately deemed to have OC and constituted the FP group. Women in the FP group evidenced a clinically and statistically significant mean 6.4 point decrease in their Mental Component Score between baseline and their most recent follow-up assessment. (Lower scores represent poorer mental QOL). Women with no abnormal OC screening test result constituted the Normal group (*n* = 83) and evidenced a statistically and clinically insignificant mean 0.67 point drop in their Mental Component Score. It was concluded for women at intermediate risk for OC, FP screening results are associated with a significant decline in mental QOL. 

As part of the United Kingdom Familial Ovarian Cancer Screening Study (OKFOCSS), women at increased genetic risk for OC (estimated lifetime risk >10%) participated in an OC screening program involving thrice yearly CA125 testing coupled with annual TVS [23]. If an abnormal test result occurred for either type of testing, women were recalled for retesting within two months. Women (*n* = 1999) completed a baseline assessment (T1) one month prior to an initial CA125 test and a follow-up assessment one week after receiving the result of this initial test (T2). Women receiving an abnormal test result at any time completed an additional follow-up one week after repeat of the screening test ruled out malignancy and they were returned to routine screening (T3). These women constituted the FP group (*n* = 167). The remaining women who received only normal test results during participation in the screening program constituted the Normal group. Finally, all women completed a final follow-up assessment nine months after receiving a normal test result (*n* = 825) or nine months after return to routine screening after receiving a FP result (*n* = 87) (T4). Specific outcomes assessed at each assessment included OC-specific distress, anxiety and depression symptoms, and reassurance. Compared to the Normal group the FP group reported moderately elevated OC-specific distress one week after being notified of their abnormal result. No difference in OC-specific distress was evident at T3 or T4, after women with FP results returned to routine screening. No differences with regard to anxiety, depression, or reassurance were noted between the Normal screening and FP groups at any assessment point. Finally, women in the FP group were significantly more likely to withdraw from the screening program (primarily for risk-reducing salpingo oophorectomy) (OR = 4.38). It was concluded FP screening results are associated with transient OC-specific distress but are not associated with sustained psychological harm. 

Finally, 111 female BRCA1/2 mutation carriers who had not undergone risk-reducing surgery (i.e., salpingo oophorectomy) participated in a screening program involving both annual breast cancer screening and OC screening involving both CA125 testing and TVS [26]. All abnormal screening test results were followed by additional imaging and biopsy when appropriate. All participants completed a questionnaire prior to a baseline screening visit, as well as 3 and 12 months post-baseline. Psychological outcomes assessed included general anxiety, perceived absolute and comparative risk for OC, and OC worry. Women receiving normal OC screening test results following baseline screening constituted the Normal group and those receiving abnormal results with no cancer subsequently detected constituted a FP group. No significant differences were found between the Normal and FP groups for any of the OC risk or worry outcomes at either the 3 or 12 month follow-ups. Furthermore, there were no differences between the two groups in the likelihood of undergoing risk-reducing surgery. It was concluded FP test results were not associated with large increases in risk perception, cancer worry, or uptake of risk-reducing surgery. Unfortunately, only two women received abnormal CA125 test results (<2% of the sample) while the number of women receiving FP TVS test results was not reported. As a result, it is unclear whether the lack of significant findings might be due to a true absence of effect or simply due to inadequate statistical power.

### 3.2. Summary: Impact of FP Results on Women at Intermediate or High Risk for OC

Each of the studies considered in this group examined the impact of a FP screening test result on one or more psychological outcomes. Overall, the evidence suggests that a FP result in screening programs targeted at women at intermediate or high risk for OC is associated with significantly poorer psychological status in the immediate aftermath of an abnormal OC screening test result. This appears particularly true with regard to cancer-specific worry and distress [23,25,27]. Little evidence suggests much impact on more general indices of depression, anxiety or mental health or on perceptions of OC risk. Any impact on psychological status appears to be short-lived, however, as differences between FP and Normal groups generally decline over time following determination no malignancy is present [23,28]. The study by Kauff et al. [25] appears to be an exception to this general conclusion, however, as they found FP results were associated with significant and long-term declines in a generic measure of mental health. However, their data analytic strategy did not account for when a FP result was experienced in the course of the mean nearly 20 months of observation. This makes it difficult to attribute any observed declines in mental health status to the experience of a FP screening test result. Finally, Brain et al. found women receiving FP results were over four times more likely to withdraw from their OC screening program [23]. Withdrawal was typically followed by risk-reducing surgery, however, eliminating the need for further OC screening. It was unclear, however, whether women in the FP group were more likely than women in the Normal group to discontinue participation in OC screening in the absence of risk-reducing surgery. This is a critical missing piece of information given any risk-reducing impact of a screening program is predicated upon continued, appropriate participation with the program [30]. 

### 3.3. Impact of FP Results on Women at Average Risk for OC

The studies considered here examined the psychological and behavioral impact of a FP OC screening test result on asymptomatic women generally at average risk for OC [20,21,22,24,29]. Andersen et al. examined women (*n* = 592) at “conventional” risk for OC (i.e., ≤1 first degree relative with OC) undergoing alternating CA125 and TVS testing every six months for 18 months [20]. Abnormal CA125 results were followed by TVS screening while abnormal TVS results required repeat TVS screening in six to eight weeks. Measures of QOL, worry about OC risk, and OC-specific distress were completed at a baseline assessment prior to the initial OC screening test and a follow-up two years post-baseline, after the screening program was concluded. At follow-up, women receiving a FP OC screening test result at some point during the two-year screening period (*n* = 32) were compared to similar women who received normal screening test results (i.e., Normal group). The FP group reported significantly greater worry about OC risk. Cancer-specific distress and quality of life did not differ between the FP and Normal screening groups. It was concluded FP screening test results may have long-term effects and increase worry about cancer risk.

Barrett et al. examined the psychological impact of a FP screening test in women participating in the United Kingdom Collaborative Trial of Ovarian Cancer Screening (UKCTOCS), a randomized trial of annual multimodal OC screening [21]. Women (*n* = 202,638) were randomized to one of three arms: (1) OC screening with CA125 testing followed, if necessary, by TVS (CA125+TVS) as a second line test; (2) OC screening with TVS; or (3) no OC screening. Following receipt of an abnormal screening test result women in the CA125+TVS arm underwent a repeat CA125 test with (Level 2) or without (Level 1) TVS testing. Women in the TVS group underwent a repeat TVS test by a senior ultrasonographer within three months (Level 1) or a repeat TVS test or biopsy within six weeks (Level 2). All women completed a baseline questionnaire prior to randomization. Women in the two screening groups who received an abnormal screening test result (*n* = 22,035) completed a questionnaire following each abnormal screen and annually thereafter. The questionnaire included measures of state anxiety as well as general psychological morbidity as assessed by the General Health Questionnaire (GHQ-12). Results indicated greater psychological morbidity in women after receipt of a FP test result but only for women receiving more intensive repeat screening (i.e., Level 2 repeat screening). In other words, women in the CA125+TVS screening group exhibited greater psychological morbidity only if repeat screening involved repeat of both the CA125 and TVS tests while women in the TVS screening group exhibited greater psychological morbidity only if repeat screening involved a TVS scan or biopsy (i.e., Level 2 repeat screening). No differences between the FP and Normal groups were found for state anxiety. It was concluded a FP test results in slightly increased psychological morbidity when an abnormal screening test result is followed by more intensive forms of repeat screening.

The remaining studies in this group examined asymptomatic, average-risk women participating in an ongoing trial of OC screening using annual TVS at the University of Kentucky [22,24,29]. In this trial, an abnormal TVS test result requires repeat of the TVS screening test in 2–16 weeks. In an initial study [22], women (*n* = 540) completed a baseline assessment immediately before undergoing a routine annual TVS screening test and follow-up assessments two weeks and four months post-baseline. A single-item measure of OC risk perception (i.e., perceived lifetime personal risk) as well as measures of general and OC-specific distress were completed at all assessments. Women receiving a FP screening test result (*n* = 33) at baseline were compared to women receiving a normal screening test result (i.e., Normal group; n = 507). Compared to women receiving a “normal” TVS test result, women receiving a FP result reported significantly elevated OC-specific distress at the two-week follow-up, before a repeat TVS test had clarified whether a malignancy was present. Distress returned to baseline levels at four-month follow-up, after repeat of the TVS test indicated no malignancy was present. No differences between the FP and Normal groups on mood disturbance, depression, or OC risk perception were found. It was concluded a FP test result is associated with a significant, but transient, increase in OC-specific distress. 

As a follow-up to this initial study, a larger longitudinal study examining psychological and behavioral responses to receipt of a FP OC screening test result was implemented [29]. Women receiving a FP TVS test result (*n* = 375) in the course of routine, annual TVS screening were compared to women (*n* = 375) receiving a normal test result (i.e., Normal group). Women in the FP group were matched with women in the Normal group with regard to age, family history of OC, and OC screening history (prior FP result, number of prior TVS tests). Women in the FP group completed a baseline assessment immediately prior to undergoing a repeat TVS test, required to clarify the nature of a recent abnormal TVS test 2 to 16 weeks earlier. Women in the Normal group completed a baseline assessment immediately prior to a routine, annual TVS screening test. Both groups completed follow-up assessments one and four months after baseline. Results indicated FP test results were associated with clinically significant increases in OC-specific distress with distress remaining elevated through the four month follow-up. Women receiving a FP result also reported significantly higher perceptions of OC risk on two different, two-item composite measures of risk (Personal OC Risk and Comparative OC Risk) and fewer perceived positive consequences of screening participation. A FP test result also impacted screening intentions as the FP group reported at one month follow-up significantly weaker intentions to return for future OC screening. No differences between the FP and Normal groups were found with regard to depressive symptoms, benefit finding, or beliefs in the effectiveness of OC screening at any of the study assessments. It was concluded FP results negatively impact both affective (OC-specific distress) and cognitive (risk perception) outcomes in both the short and intermediate term. The negative psychological impact of a FP test is still evident for several months after repeat testing has ruled out malignancy and may serve to reduce motivation to participate in future routine OC screening. 

Finally, Floyd et al. examined the impact of a FP test result on women’s interest in receiving health-related information [24]. Based on the possibility a FP screening test result could be experienced as a very scary and threatening event [1], it was hypothesized a FP result may serve as a “teachable moment” [31] and enhance interest in obtaining additional information about OC as well as other health-related topics. A Normal screening group (*n* = 124) consisted of women undergoing a routine, annual TVS test with receipt of normal results. This group completed a baseline assessment prior to undergoing routine TVS screening. A FP screening group (*n* = 279) consisted of women who had received an abnormal TVS test result 2–16 weeks earlier and were returning for a repeat TVS test to clarify whether a malignancy was present. These women completed a baseline assessment prior to undergoing this repeat TVS screening test. All women completed a follow-up assessment one month post-baseline where interest in receiving information about 10 different health- and cancer-related topics was assessed. Contrary to hypothesis, results indicated women receiving FP screening test results were significantly less interested in receiving health- and cancer-related information than women receiving normal screening results. It was concluded receipt of a FP result does not represent a teachable moment when women may be more receptive to health information and health behavior change. Rather receipt of a FP screening test result appears to make women less interested in health-related information, including information regarding OC. 

### 3.4. Summary: Impact of FP Results on Women at Average Risk for OC

Most studies in this group examined the impact of a FP screening test result on one or more psychological outcomes [20,21,22,29]. In each of these studies, a negative impact on psychological outcomes was observed. Receipt of a FP result was associated with poorer psychological status, particularly OC-specific worry [20] and OC-specific distress [22,29]. Little evidence suggests FP results exert a significant impact on more general measures of psychological morbidity or distress, although one study did find elevated psychological morbidity in women receiving a FP result requiring more intense, invasive repeat testing after an abnormal result [21]. While it is clear FP results impact OC-specific distress, the duration of this impact is not completely clear. It is quite apparent receipt of an abnormal screening test result is associated with an immediate and pronounced spike in OC-specific distress [22,29] which remains elevated for at least several weeks or months even after repeat OC screening rules out malignancy [29]. Overall, the evidence suggests that receipt of a FP screening test result increases OC-specific distress and worry in the short term and quite possibly in the intermediate term. As for other outcomes, the data are sparse or mixed. The two studies that examined the impact of a FP test result on perceptions of OC risk yielded mixed results [22,29]. While one study found no impact upon OC risk perception [22], a larger and better designed study found a FP result resulted in higher perceptions of OC risk up to four months after repeat TVS testing ruled out malignancy [29]. The failure to find a significant impact on OC risk perception in the earlier study [22] could be due to a lack of statistical power as well as use of a relatively crude measure of OC risk perception. The earlier study [22] included only 33 women in the FP result group compared to 375 women in the later study [29], making it less likely a “true” impact on OC risk perception could be detected in the earlier study. Additionally, the earlier study indexed OC risk perception using only a single, single-item measure of risk perception. In contrast, the later study [29] found significant effects on two separate, two-item composite measures of OC risk perception. Finally, a FP result may impact the likelihood of participation in future screening as receipt of a FP result was associated with significantly weaker intentions to return for future OC screening [29]. 

## 4. Impact of Normal OC Screening Test Results

As might be expected, an abnormal or FP test result appears to have at least some negative impact on psychological and behavioral outcomes. Might the opposite be true? Does receipt of a “normal” OC screening test result yield a positive impact on psychological or behavioral outcomes? In contrast to the attention focused upon the impact of abnormal or FP results, it appears the impact of a “normal” test result on psychological and behavioral outcomes has received less attention. In an initial study, high-risk women (*n* = 275) underwent ultrasound screening for OC [32]. Nearly ¾ reported feeling reassured after screening. In a more recent and comprehensive study, asymptomatic, average-risk women (*n* = 560) completed a baseline assessment immediately prior to undergoing routine, annual TVS screening for OC as well as follow-up assessment two weeks and four months post-baseline [33]. All women received a “normal” screening test result. Growth curve modeling revealed receipt of a “normal” test result was associated with a significant decrease in OC-specific distress and significant increases in positive affect, belief in the efficacy of OC screening, and knowledge of OC risk factors between the baseline and four-month follow-up assessment. No effect was observed on OC risk perceptions, negative affect, or more generic measures of distress (i.e., depression, mood disturbance). It was concluded participation in routine OC screening with receipt of a normal result can positively impact affective and cognitive psychological outcomes that can serve to promote continued participation in OC screening. 

## 5. Summary and Recommendations

Determination of the value of any cancer screening test requires careful consideration of the costs and benefits associated with that screening test. Obviously, certain types of costs and benefits are weighed more heavily than others. The paramount benefit of a screening test is the extent to which it reduces cancer-related mortality. On the cost side of the ledger, the economic and physical morbidity costs (e.g., surgeries) associated with a screening test are weighed most heavily. Less frequently considered are any psychological and behavioral costs and benefits associated with a cancer screening test. 

The purpose of this paper was to consider the psychological and behavioral impact, both costs and benefits, associated with participation in OC screening. The studies we considered are diverse in methodology and design. Most of these studies focused upon documenting the psychological “costs” associated with abnormal, ultimately FP, OC screening test results. In general, these studies clearly suggest that women who experience a FP OC screening test result report more OC-specific worry and distress in the short-term [22,23,27,29]. This is unsurprising, of course, given receipt of an abnormal result raises at least the possibility of a subsequent diagnosis of OC. This distress appears to dissipate over time, but may still be present to a degree four months to a year or more after receipt of an abnormal result and after clinical follow-up has ruled out a malignancy [20,28,29]. This general conclusion appears to be true regardless of the OC risk status of the asymptomatic women being screened. 

In contrast to the findings for OC-specific distress and worry, the impact of a FP screening test result on other psychological outcomes is less clear. The impact on generic measures of mental health, depression and anxiety is mixed with some research showing a negative impact [21,25,27] and other research showing no impact [20,22,23,29]. Similarly, the impact of a FP result on perceptions of OC risk is also mixed with some studies finding no impact [22,27] but another, larger, better-designed study finding increased perceptions of OC risk in women experiencing a FP result [29].

With regard to behavioral costs associated with a FP OC screening test result, FP test results may be associated with reduced future participation in OC screening [23] or reduced intentions to return for routine screening in the future [29]. Given the effectiveness of any cancer screening modality is predicated upon continued screening uptake at appropriate intervals, this is a significant potential cost associated with FP results in the OC screening setting.

In contrast to the negative impact of a FF OC screening test result, it appears woman may benefit from participation in routine OC screening when a “normal” screening test result is received. For asymptomatic, average-risk women, participation in OC screening with receipt of a “normal” test result was associated with a significant decrease in OC-specific distress and significant increases in positive affect, belief in the efficacy of OC screening, and knowledge of OC risk factors over a four month period following screening [33]. Additionally, comparison of women receiving FP and normal results found women receiving normal screening test results attributed more positive consequences to their screening experience such as greater feelings of well-being and reassurance [29]. This data is provocative in its suggestion that participation in screening may not be a completely benign experience from a psychological and behavioral standpoint. Rather, participation in screening may create affective and cognitive conditions that may not only be inherently positive and reinforcing, but may also serve to further promote continued participation in OC screening. 

While showing some ability to detect OC at an earlier stage, randomized trials have failed to show a reduction in cancer-specific mortality in asymptomatic, average-risk women participating in OC screening programs. Coupled with consideration of the monetary costs of OC screening and the potential physical morbidity costs (e.g., surgery) associated with FP test results, the US Preventive Services Task Force recommends against routine screening for OC in asymptomatic, average-risk women (D recommendation) [17]. Despite this negative recommendation, routine screening of asymptomatic women at average risk for OC is unlikely to go away. OC screening is well accepted by many physicians [18] as well as by the public. An overwhelming majority of women participating in the University of Kentucky OC screening program indicated they agreed or strongly agreed that TVS screening is effective in the early diagnosis of OC [29]. As a result, further research on the psychological and behavioral impact of participation in OC screening is warranted. While more research is needed, what is particularly needed is better research. Future research examining the impact of FP screening test results should be characterized by (1) sufficient numbers of women receiving abnormal or FP results to ensure adequate statistical power and the ability to interpret null findings; (2) longitudinal assessment to enable identification of the duration of impact of a specific abnormal or FP test result on key psychological and behavioral outcomes; and (3) removal of women who undergo risk-reducing oophorectomy from analyses focused upon understanding the impact of FP results on future screening participation. 

In addition, there is likely benefit from expanding the focus of research regarding the psychological and behavioral impact of participation in OC screening. In particular, more research is needed regarding the potential impact of participation in routine OC screening with receipt of a “normal” test result. While research has examined the potential positive impact of a normal test result [33] the potential negative impact of a normal test result has received scant, if any, attention. Potential negative impact might involve delay in help-seeking following any subsequent onset of symptoms related to OC or a general complacency about health. Two other areas are also largely unexamined: the psychological and behavioral impact of false negative OC screening test results—receipt of a normal screening test result when OC is present—and the impact of true positive OC screening test results. In the latter case, the psychological and behavioral impact of a diagnosis of OC may differ depending on whether OC was screening- or symptomatically-detected. 

In conclusion, the research considered here does not suggest that FP test results in the course of screening asymptomatic women for OC result in significant, durable psychological harm. In addition, there is some suggestion that participation in routine screening for OC may confer psychological benefits. Some have contended OC screening “does more harm than good” [34]. If true, this contention is true based largely on consideration of the physical harms associated with surgeries performed for diagnostic or preventive purposes following abnormal OC screening results. Any psychological or behavioral harms attributable to OC screening appear to be, at worst, rather modest in severity and duration and might well be counterbalanced by psychological benefits accruing to women who participate in routine OC screening and who receive normal test results. 
